# A Widespread Bacterial Secretion System with Diverse Substrates

**DOI:** 10.1128/mBio.01956-21

**Published:** 2021-08-17

**Authors:** Alex S. Grossman, Terra J. Mauer, Katrina T. Forest, Heidi Goodrich-Blair

**Affiliations:** a University of Tennessee—Knoxville, Department of Microbiology, Knoxville, Tennessee, USA; b University of Wisconsin—Madison, Department of Bacteriology, Madison, Wisconsin, USA; Brigham and Women’s Hospital/Harvard Medical School

**Keywords:** DUF560, HACEK, HrpB, NilB, Slam, *Steinernema*, TbpB, *Xenorhabdus*, hemophilia, lipoprotein, network, outer membrane

## Abstract

In host-associated bacteria, surface and secreted proteins mediate acquisition of nutrients, interactions with host cells, and specificity of tissue localization. In Gram-negative bacteria, the mechanism by which many proteins cross and/or become tethered to the outer membrane remains unclear. The domain of unknown function 560 (DUF560) occurs in outer membrane proteins throughout *Proteobacteria* and has been implicated in host-bacterium interactions and lipoprotein surface exposure. We used sequence similarity networking to reveal three subfamilies of DUF560 homologs. One subfamily includes those DUF560 proteins experimentally characterized thus far: NilB, a host range determinant of the nematode-mutualist Xenorhabdus nematophila, and the surface lipoprotein assembly modulators Slam1 and Slam2, which facilitate lipoprotein surface exposure in Neisseria meningitidis (Y. Hooda, C. C. Lai, A. Judd, C. M. Buckwalter, et al., Nat Microbiol 1:16009, 2016, https://doi.org/10.1038/nmicrobiol.2016.9; Y. Hooda, C. C. L. Lai, T. F. Moraes, Front Cell Infect Microbiol 7:207, 2017, https://doi.org/10.3389/fcimb.2017.00207). We show that DUF560 proteins from a second subfamily facilitate secretion of soluble, nonlipidated proteins across the outer membrane. Using *in silico* analysis, we demonstrate that DUF560 gene complement correlates with bacterial environment at a macro level and host association at a species level. The DUF560 protein superfamily represents a newly characterized Gram-negative secretion system capable of lipoprotein surface exposure and soluble protein secretion with conserved roles in facilitating symbiosis. In light of these data, we propose that it be titled the type 11 secretion system (TXISS).

## INTRODUCTION

All plants and animals exist in association with bacterial symbionts that contribute to nutrition, protection, development, and reproduction. These symbionts express surface and secreted proteins that facilitate host interactions through a variety of functions, including acquisition of nutrients (1, 2), interaction with host cells (3), and specificity in host range and tissue localization (4). Possibly due to the complexity of bacterial membranes and the breadth of biophysical characteristics of secreted proteins, there is no singular export pathway.

Bacteria have two broadly distributed export systems, including the inner membrane spanning Sec and twin arginine translocation (Tat) systems which are shared between Gram-positive and Gram-negative bacteria. Diderms require additional secretion systems for transport into and across the outer membrane. The type I, II, V, IX, and X secretion systems work to transport substrates across that outer membrane, while types III, IV, and VI go one step further, moving effector proteins across the outer membrane and directly into another organism (5–7). Despite the many secretion systems described thus far, there are still a number of secreted proteins which are known to contribute to symbiosis but for which no transport system is known. Here we describe a machinery present throughout the phylum *Proteobacteria* that is responsible for secreting soluble proteins and lipoproteins from the periplasm across the outer membrane.

Recently, the Slam (surface lipoprotein assembly modulator) mechanism of lipoprotein surface tethering was identified in Neisseria meningitidis (8, 9). Slam proteins containing the β-barrel DUF560 (domain of unknown function 560) (also termed SlipAM domain) (10) are required for surface presentation of certain lipoproteins, including those that capture metal-carrying compounds used by hosts to sequester nutrients (8). Two N. meningitidis Slam proteins have been characterized, with distinct lipoprotein substrates which contribute to symbiosis. Slam activity also has been demonstrated for DUF560 representatives from pathogens Pasteurella multocida, Moraxella catarrhalis, and Haemophilus influenzae (8, 9). However, most Slam homologs have no bioinformatically predicted substrate thus far, and one study has found that N. meningitidis Slam1 can surface expose unprocessed factor H binding protein (fHbp) single nucleotide polymorphism (SNP) variants (11). Thus, the full functional potential of DUF560 proteins is not yet known.

The DUF560 homolog NilB is a host association and species specificity factor in the nematode symbiont Xenorhabdus nematophila, a proteobacterium in the family *Morganellaceae* (12–14). A screen for X. nematophila mutants defective in colonizing Steinernema carpocapsae intestines revealed the nematode intestinal localization (*nil*) locus (14, 15). The *nil* locus contains the genes *nilB* and *nilC*, each of which is independently necessary for colonization of nematodes. Biochemical and bioinformatic analyses have established that NilC is an outer membrane-associated lipoprotein, and NilB is an outer membrane β-barrel in the DUF560 family with an ∼140-amino-acid periplasmic N-terminal domain that contains tetratricopeptide repeats (15–18).

**FIG 1:**
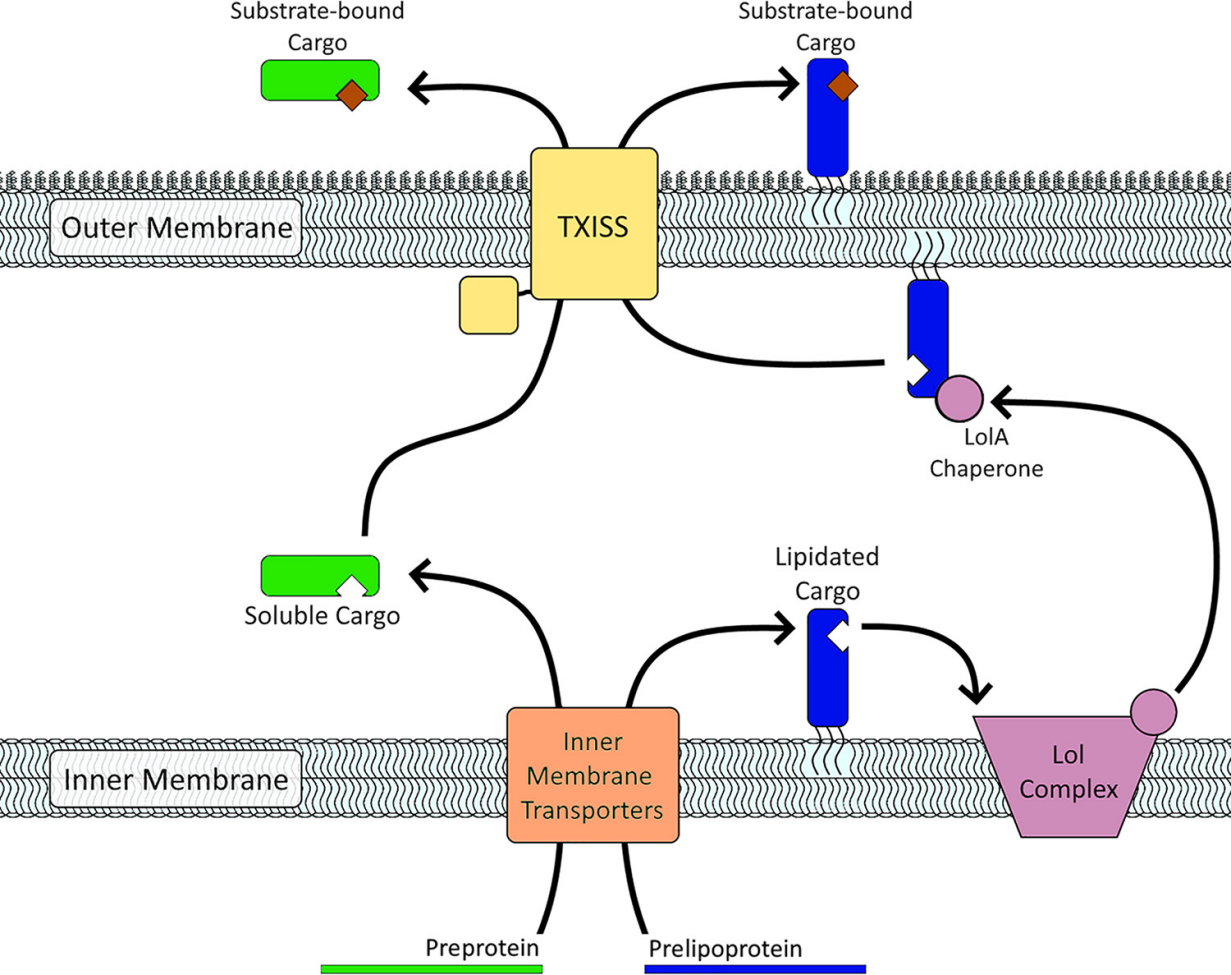
Conceptual model of the type XI secretion system. TXISS outer membrane proteins (yellow) of the DUF560 family are necessary and sufficient to secrete lipidated (blue) and soluble (green) cargo proteins across the outer membrane to the extracellular milieu. TXISS-dependent cargo proteins are exported into the periplasm via inner membrane transporters (orange). Once in the periplasm, lipidated TXISS cargo proteins, such as TbpB, are expected to be chaperoned across the hydrophilic periplasm by the localization of lipoproteins (Lol) complex. It is currently unknown whether periplasmic soluble TXISS cargo proteins have dedicated molecular chaperones to reach the outer membrane. Once secreted by TXISS, cargo proteins can bind to their specific substrates.

To begin to understand the functional range of DUF560 proteins, we assessed ecological distribution, genomic context, and relatedness. We experimentally examined the X. nematophila DUF560 homolog HrpB, which is predicted to transport an unlipidated protein. Finally, to better understand the potential role of DUF560 proteins in host-symbiont interactions, we analyzed distribution among symbiotic *Xenorhabdus*. Our data demonstrate that biological activities of the DUF560 family extend beyond lipoprotein surface presentation and constitute the type 11 bacterial secretion system (TXISS) which, like the type II secretion system, is capable of acting on membrane-anchored or soluble proteins (Fig. 1) (19).

(This article was submitted to an online preprint archive [20].)

## RESULTS

### TXISS cluster according to environment.

**FIG 2:**
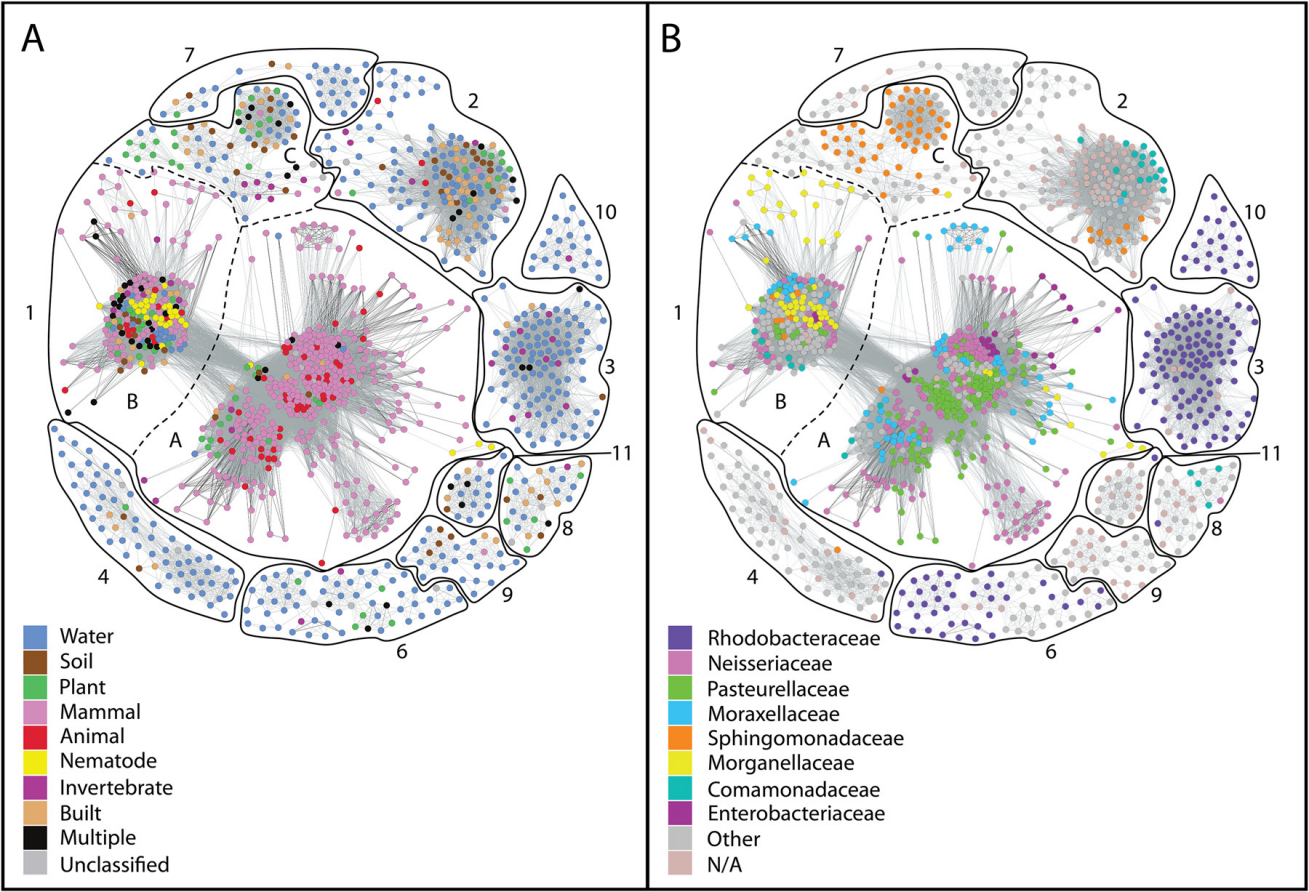
Sequence similarity network (SSN) of all DUF560 proteins. Network generated by EFI-EST as accessed on 24 April 2019 (1–3). Edges were drawn using an alignment score of 38, and any sequences which shared ≥40% identity were placed in a single node to allow the separation of clusters. Each node represents a group of highly similar sequences, with edge darkness demonstrating similarity, and the distance between nodes being determined via the Fruchterman-Reingold algorithm to optimize edge lengths (59). Each node was color coded to show the isolates’ environmental origin(s) (A) and taxonomic class (B). N/A, not applicable.

**FIG 3:**
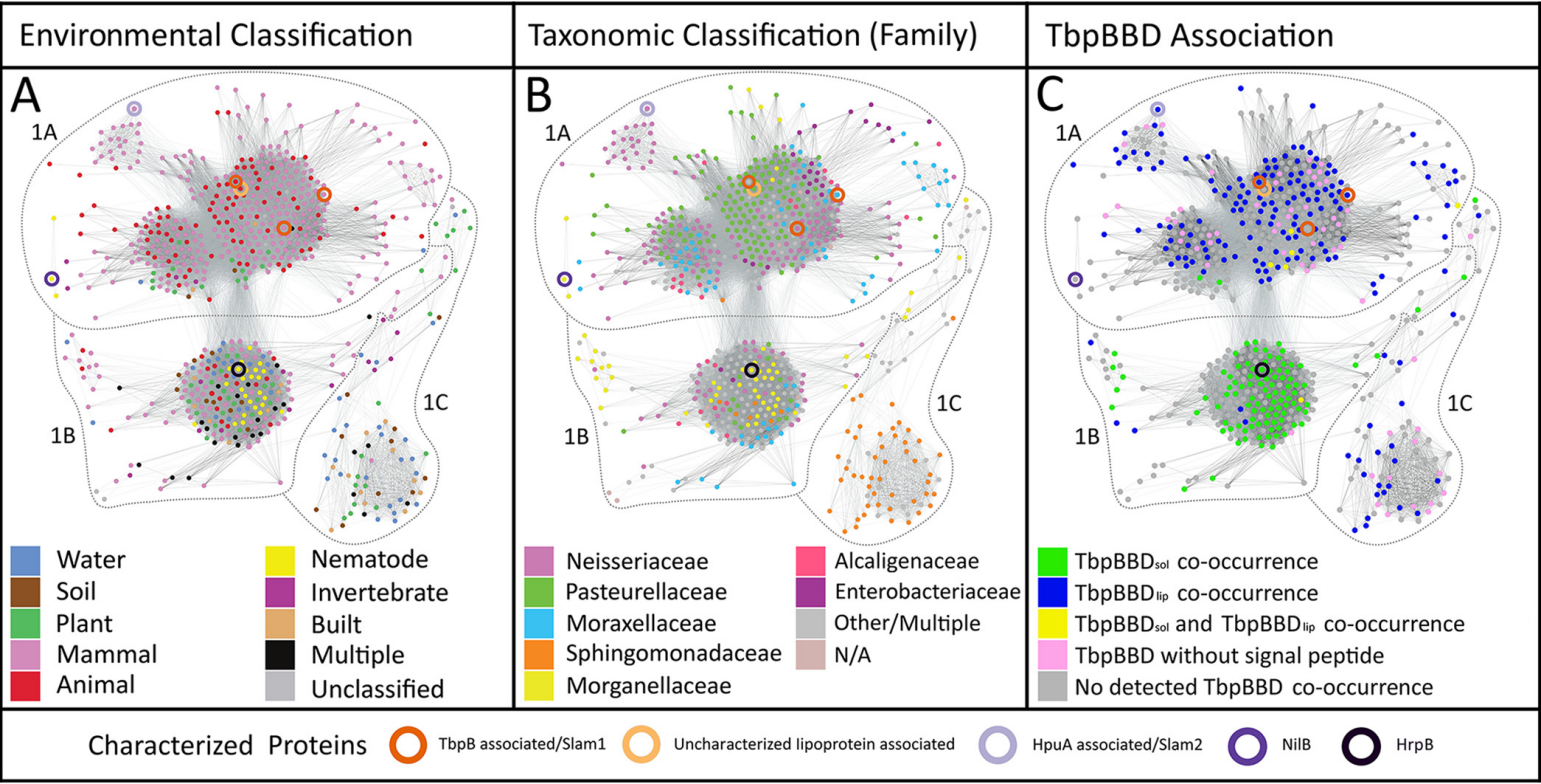
Cluster 1 of the TXISS SSN forms subclusters according to environment of isolation and signal sequence of predicted cargo. All nodes from cluster 1 of the TXISS SSN were positioned using the Fruchterman-Reingold algorithm. (A to C) The resulting graph was annotated according to environmental origin(s) (A), taxonomic class of the isolates (B), or whether the node homolog(s) cooccur with a TbpBBD domain-encoding gene (C). Nodes containing experimentally characterized proteins are highlighted using colored circles as indicated at the bottom of the figure.

Using homology to NilB or Slam proteins, previous work identified a wide distribution of DUF560 proteins within mucosa-associated bacteria (9, 14, 15). To quantifiably delineate subfamilies within the TXISS, we generated a sequence similarity network (SSN) using the Enzyme Function Initiative toolset (EFI) (21–23) and annotated it to highlight environmental source or taxonomic grouping of microbes containing DUF560 homologs (Fig. 2; see also Table S1 in the supplemental material). In this analysis, sequences with high identity (≥40%) were gathered into nodes and connected with edges based on sequence similarity. Using all homologs in Interpro 73 and UniProt 2019-02, we identified 10 major clusters of TXISS proteins. Cluster 1 was chosen for in-depth analysis as it contained the majority of nodes in the network (62.4%) and could be clearly divided into three subclusters (1A, 1B, and 1C) using force-directed node placement (Fig. 3). The remaining nine clusters displayed a preponderance of water- and soil-associated organisms and contained no characterized proteins (see Table S1 for details). Consistent with our previous observations (15), the majority of cluster 1 nodes (75%) comprise sequences from animal-associated isolates, while another 20% contain sequences from marine, freshwater, soil, or built-environment isolates.

10.1128/mBio.01956-21.6 TABLE S1 DUF560 sequence similarity network clusters 1 to 4 and 6 to 11 node table, with subcluster, environmental annotations, and sources (when possible). Download Table S1, XLSX file, 0.5 MB. Copyright © 2021 Grossman et al. 2021 Grossman et al. https://creativecommons.org/licenses/by/4.0/ This content is distributed under the terms of the Creative Commons Attribution 4.0 International license.

The division of cluster 1 nodes strongly reflected environmental origin (Fig. 3A). Subcluster 1A almost exclusively comprises animal-associated bacteria and contains all previously characterized TXISS, which separate according to the predicted substrate when analyzed with higher stringency (see Fig. S1 in the supplemental material). Subclusters 1B and 1C have no previously characterized representatives. Subcluster 1B contains a mixture of sequences from host-associated and free-living bacteria, while subcluster 1C contains sequences largely from environmentally isolated *Sphingomonadaceae*. Subclusters 1A and 1B correlate poorly with taxonomy (Fig. 3B). For example, cluster 1 contains 81 nodes with *Moraxellaceae* sequences. Of these, 79% are animal associated and are enriched in subcluster 1A, while 12.3% are environmental isolates and are enriched in subcluster 1B. These data demonstrate a correlation with lifestyle (e.g., free-living versus host associated) as opposed to taxonomy and suggest that subclusters indicate divergent functions.

10.1128/mBio.01956-21.1 FIG S1 Increased stringency separates subclusters 1A and 1B into functional groups. A series of stringent EFI-EST sequence similarity networks highlights detail in cluster 1 of the DUF560 homologs. Edge darkness demonstrates similarity. Node positioning was optimized using the Fruchterman-Reingold algorithm (59). Dotted lines indicate hypothetical functional clusters based on previous molecular data. Circled nodes indicate proteins that have been molecularly characterized. Subclusters 1A and 1B were analyzed separately to allow fine tuning of alignment score (89 and 100, respectively). Networks were color coded to display either taxonomic categories or coinheritance with TbpBBD domain-containing proteins. Download FIG S1, PDF file, 0.5 MB. Copyright © 2021 Grossman et al. 2021 Grossman et al. https://creativecommons.org/licenses/by/4.0/ This content is distributed under the terms of the Creative Commons Attribution 4.0 International license.

### TXISS cluster according to substrate.

The Slam acronym was defined on the basis that DUF560 homologs from N. meningitidis facilitate surface exposure of lipoproteins, such as transferrin binding protein B (TbpB), lactoferrin binding protein B (LbpB), hemoglobin/haptoglobin binding protein A (HpuA), and fHbp, which are frequently encoded nearby (8, 9). Yet the lipid tail is not essential for Slam-dependent surface exposure of a target (8, 11, 24), prompting us to consider whether cooccurrence with lipoproteins is a constant. We used the EFI Genome-Neighborhood Tool (21, 23, 25) to assay the genomic context of cluster 1 members. This analysis corroborated previous work demonstrating genomic association of DUF560 proteins with TonB, TonB-dependent receptors, and proteins that have a Pfam TbpB_B_D domain, which will be referred to hereafter as TbpBBD (9, 26).

**FIG 4:**
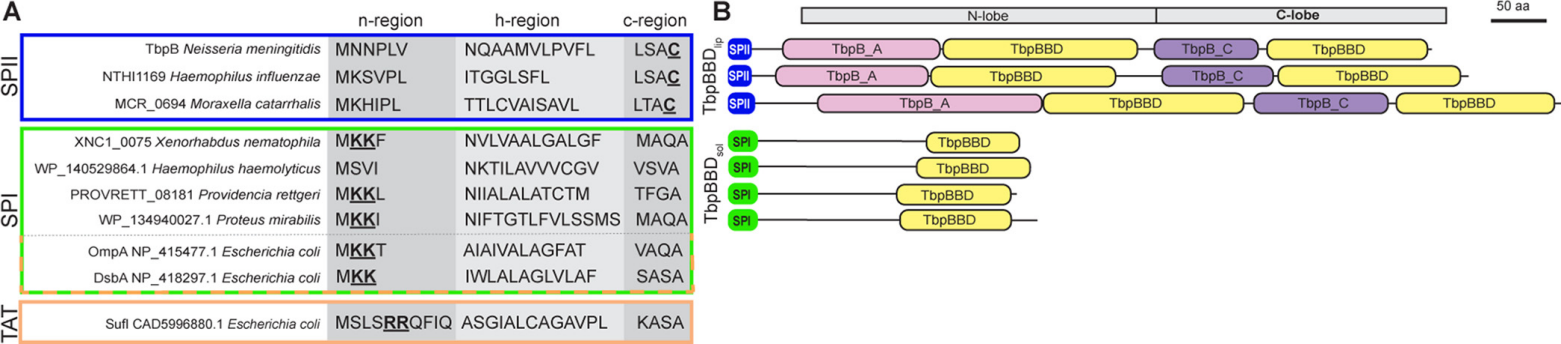
Examples of TbpBBD_lip_ and TbpBBD_sol_ signal peptides and domain architectures. TXISS-1A-associated proteins from N. meningitidis, H. influenzae, and M. catarrhalis have signal peptidase 2 (SPII; blue) signal sequences, while TXISS-1B associated proteins from *X. nematophila*, *H. hemolyticus*, *P. rettgeri*, and P. mirabilis have signal peptidase 1 (SPI; green) signal sequences. (A) SPII and SPI signal sequences, comprising n-, h-, and c-regions of representative TXISS-1A (blue) and TXISS-1B (green, above dashed line) cargo proteins and two E. coli proteins (OmpA and DsbA) that can be secreted through either the Sec or Tat machinery (below dashed line, green-orange border), are compared to a canonical Tat secretion signal (orange) found in the E. coli protein SufI. Underlined amino acids highlight conserved features of signal sequences, including the acylated cysteine of lipoproteins, the twin arginine motif of Tat signal peptides, and n-region twin lysines that are permissive for Tat secretion (28). (B) The schematic diagram shows features found in select TbpBBD domain-containing proteins predicted to be exported by TXISS mechanisms. TXISS-1A-associated cargo proteins shown have SPII (blue), N-lobe and C-lobe handle domains TbpB_A (pink) and TbpB_C (purple), respectively, and two TbpBBD domains (yellow). TXISS-1B-associated cargo proteins shown have SPI (green), lack annotated handle domains, and have a single TbpBBD domain (yellow).

Given the prevalence of TbpBBD domain-containing genes in the neighborhoods of DUF560 genes, and their known occurrence in lipoproteins exposed by Slams, we examined their open reading frames (ORFs) for potential patterns. Using a combination of genome-neighborhood-network data (23), Rapid ORF Description & Evaluation Online (RODEO) data (27), and manual annotation, we analyzed 851 unique TbpBBD domain proteins coinherited with cluster 1 DUF560s (Table S2). The majority (75.1%) of TbpBBD-bearing proteins associated with subcluster 1A have a signal peptidase 2 (SPII) signal peptide, indicating lipidation, and two TbpBBD domains, similar to TbpB in N. meningitidis (9). These are referred to here as TbpBBD_lip_ (lipidated TbpBBD). In contrast, the TbpBBD-bearing proteins associated with subcluster 1B are almost exclusively predicted to be soluble proteins (97.8%) with signal peptidase 1 (SPI) signal peptides and a single TbpBBD domain, similar to hemophilin in Haemophilus haemolyticus. Hereafter we refer to this class of proteins as TbpBBD_sol_ (for soluble TbpBBD) (Fig. 3C and Fig. 4). TbpBBD_lip_ and TbpBBD_sol_ proteins are predicted to be translocated across the inner membrane through the Sec secretion pathway. However, at least some TbpBBD_sol_ proteins have similarities to Escherichia coli OmpA and DsbA, which can be secreted through either the Sec or Tat secretion pathways (Fig. 4), leaving open the possibility that some TXISS-1B TbpBBD_sol_ proteins are conditionally Tat secreted (28).

10.1128/mBio.01956-21.7 TABLE S2 DUF560 TbpBBD protein co-occurrence. Download Table S2, XLSX file, 0.2 MB. Copyright © 2021 Grossman et al. 2021 Grossman et al. https://creativecommons.org/licenses/by/4.0/ This content is distributed under the terms of the Creative Commons Attribution 4.0 International license.

Biochemical and structural evidence support the conclusion that the TbpBBD_sol_ protein hemophilin is a soluble secreted protein that binds free heme and facilitates heme uptake (29). Three-dimensional homology modeling (Phyre^2^) was used to visualize potential structural similarities between hemophilin and several TbpBBD_sol_ proteins: a previously described homolog *X. nematophila* XNC1_0075 and two of its most closely related homologs, Providencia rettgeri PROVRETT_08181 and Proteus mirabilis WP_134940027.1 (15, 30–32) (Fig. S2). No high confidence models were found for the first ∼50 residues, which correspond to the position of the variable N-terminal handle domains of TbpBBD_lip_ proteins. However, the structures of the central regions and the C-terminal TbpBBD β-barrel domains were predicted with high confidence (>99%) based on hemophilin. In light of sequence and structural level similarities, we predict two functional TXISS subfamilies; TXISS-1A members flip TbpBBD_lip_ substrates across the outer membrane, and TXISS-1B members secrete TbpBBD_sol_ substrates into the extracellular milieu.

10.1128/mBio.01956-21.2 FIG S2 Phyre^2^ models of select TbpBBD_sol_ proteins. TbpBBD_sol_ proteins, lacking the signal sequence (-SS), from *X. nematophila* (HrpC), *P. rettgeri* (PROVRETT_08181 and 05852), and P. mirabilis (WP_134940027.1) were queried through the Phyre^2^ Protein Homology/analogy Recognition Engine v. 2.0 (http://www.sbg.bio.ic.ac.uk/phyre2/html/page.cgi?id=index) (32). The top predicted structural model output for each is shown alongside the solved crystal structure of hemophilin from H. haemolyticus (Protein Data Bank file 6OM5) which the algorithm selected as the template for all queries (29). PDB files were visualized with Protean 3D v15. (Protean 3D version 15.0; DNASTAR, Madison, WI). Download FIG S2, PDF file, 0.2 MB. Copyright © 2021 Grossman et al. 2021 Grossman et al. https://creativecommons.org/licenses/by/4.0/ This content is distributed under the terms of the Creative Commons Attribution 4.0 International license.

### TXISS-1B activity reconstructed *in vivo*.

**FIG 5:**
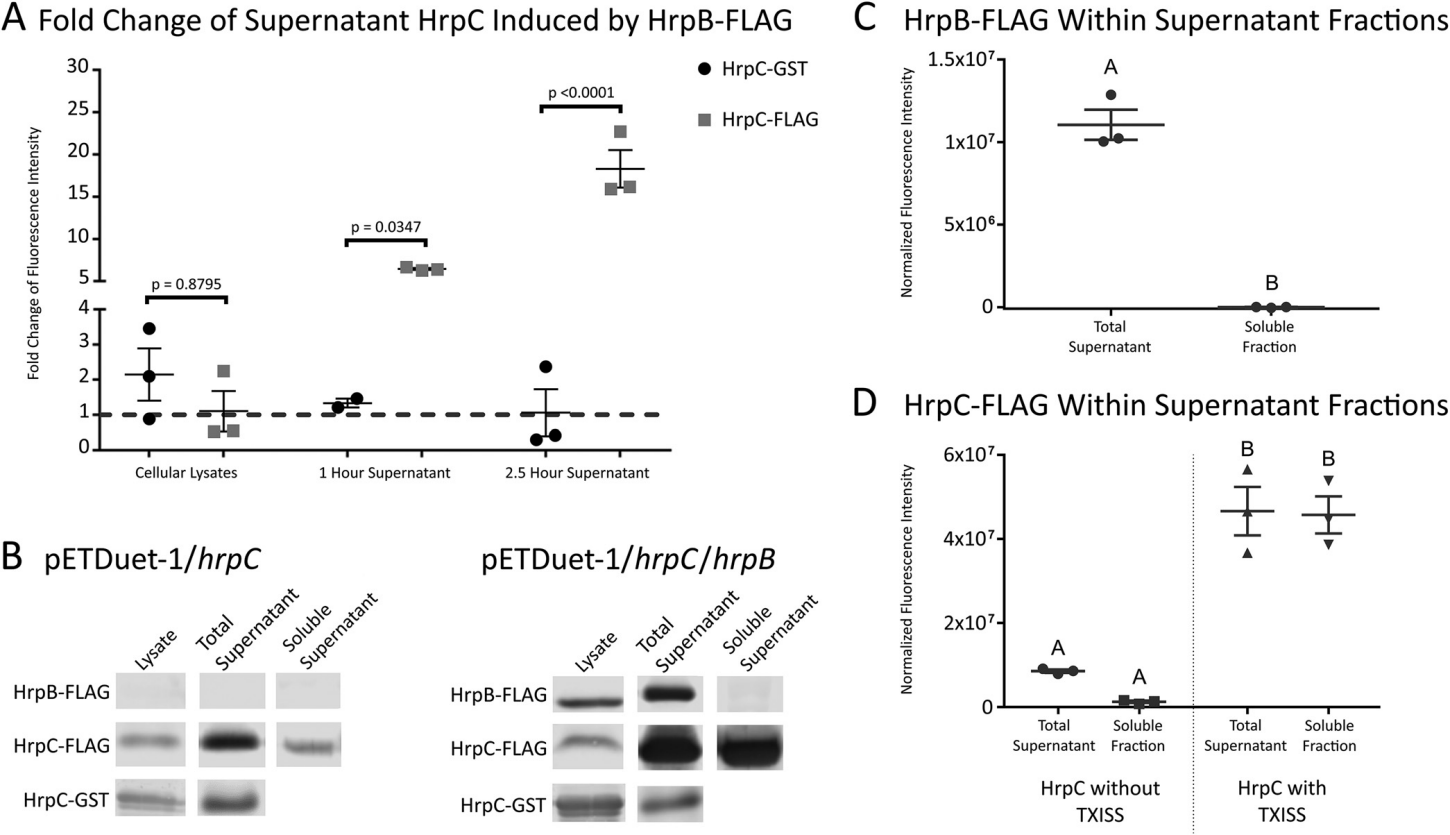
HrpB increases secretion of HrpC. (A) Demonstration of HrpB-dependent secretion. HrpC was detected and quantified as fluorescence intensity observed in immunoblots with anti-tag (FLAG or GST) antibodies (see Fig. S3 for complete blots). Fold change values shown are the fluorescence intensity of HrpC coexpressed with HrpB, divided by the intensity of HrpC expressed alone as observed from immunoblots. Each dot represents fold change derived from a distinct biological replicate pair and standard error is shown. At both assayed time points, the total supernatant concentration of HrpC-FLAG was increased by coexpression with FLAG-HrpB compared to the HrpC-GST treatment which was seemingly unaffected by the presence of FLAG-HrpB (unpaired *t* test). (B) Representative immunoblots comparing FLAG-HrpB, HrpC-FLAG, and HrpC-GST in cellular and supernatant fractions. (C) Fluorescence intensity of HrpB in supernatant fractions normalized to the Coomassie blue loading control. Each dot represents a distinct technical replicate. HrpB is not present in the soluble fraction, suggesting that it is likely in OMVs (unpaired *t* test). (D) Fluorescence intensity of HrpC-FLAG in supernatant fractions normalized to the Coomassie blue loading control. Each dot represents a distinct technical replicate pair. The TXISS-secreted HrpC is mostly located in the soluble fraction (Tukey’s HSD test).

To experimentally evaluate our prediction that TXISS-1B can secrete TbpBBD_sol_ substrates, we investigated the heme receptor protein (Hrp) locus of *X. nematophila.* This locus, conserved across the *Xenorhabdus* genus, encodes TonB, a TonB-dependent heme receptor HrpA (XNC1_0073), the TXISS-1B homolog HrpB (XNC1_0074), and its predicted TbpBBD_sol_ substrate HrpC (XNC1_0075) (15), the hemophilin homolog described above (29). Specifically, we sought to test whether HrpB mediates secretion of HrpC. Immunotagged HrpC was expressed with or without immunotagged HrpB in E. coli. Whole-cell and culture supernatant fractions were separated and analyzed by immunoblotting with anti-FLAG antibody (33). Whole-cell lysates demonstrated equivalent expression of HrpC in all treatments (Fig. 5A). In the presence of FLAG-tagged HrpB (FLAG-HrpB), the levels of HrpC-FLAG detected in culture supernatants increased 9.9-fold at 1 h postinduction and 17.0-fold at 2.5 h postinduction (Fig. 5A and B), demonstrating that secretion of HrpC is mediated by HrpB.

10.1128/mBio.01956-21.3 FIG S3 Western blots and Coomassie blue-stained SDS-PAGE gels demonstrating secretion. Units to the left of each blot/gel are in kilodaltons (kDa). All experiments were repeated in triplicate as labeled above the lanes. Orange boxes in an immunoblot indicate either FLAG-HrpB, HrpC-FLAG, or HrpC-GST. Orange boxes on a Coomassie blue-stained blot/gel indicate the bands used for normalization of fluorescent intensity values. The top row shows Western blots of FLAG-HrpB/HrpC-FLAG coexpression probed with anti-FLAG antibody and accompanying Coomassie blue-stained SDS-PAGE gels. The ∼50-kDa bands show FLAG-HrpB, while the ∼30-kDa bands show HrpC-FLAG. Expression of HrpB did not cause cell lysis or nonspecific protein export, if it had the banding pattern of the supernatant would look more like that of the lysates. The second row shows Western blots of FLAG-HrpB/HrpC-GST coexpression probed with anti-GST antibody and Coomassie blue-stained SDS-PAGE gels. The ∼55-kDa bands show mature HrpC-GST, while the lower bands are likely degradation products. The third row shows Western blots of FLAG-HrpB/HrpC-FLAG supernatant fractions probed with anti-FLAG antibody and the accompanying Coomassie blue-stained SDS-PAGE gels. The ∼50-kDa FLAG-HrpB band is removed by ultracentrifuging the supernatants, demonstrating that the HrpB is likely in OMVs. The fourth row depicts a Western blot of the FLAG-HrpB/HrpC-FLAG OMV fraction probed with anti-FLAG antibody and the accompanying Coomassie blue-stained SDS-PAGE gel. OMVs can contain both FLAG-HrpB and HrpC-FLAG. Download FIG S3, PDF file, 2.8 MB. Copyright © 2021 Grossman et al. 2021 Grossman et al. https://creativecommons.org/licenses/by/4.0/ This content is distributed under the terms of the Creative Commons Attribution 4.0 International license.

A trivial explanation for these data could be that HrpB expression leads to cell lysis. The supernatant protein profile revealed by Coomassie blue staining of sodium dodecyl sulfate-polyacrylamide gels (SDS-PAGs) did not indicate cell lysis or nonspecific protein secretion (Fig. S3). To further rule out this possibility, we created an HrpC−glutathione *S*-transferase (GST) fusion protein and coexpressed it with FLAG-HrpB. Previous observations had demonstrated that GST-fused TbpB is not surface exposed by Slam1 (8). Consistent with this finding, the levels of HrpC-GST in a culture supernatant were unaffected by coexpression with FLAG-HrpB, supporting the conclusion that expression of the outer membrane protein HrpB does not cause bacterial lysis (Fig. 5A and B) and moreover demonstrating that HrpB cannot secrete its substrate when HrpC is fused to a 26-kDa protein.

In the absence of Slam1, a fraction of the cargo protein fHbp is surface exposed when expressed in E. coli BL21(DE3) (34, 35). Similarly, we found that some HrpC-FLAG and HrpC-GST reached the supernatant in the absence of FLAG-HrpB. Furthermore, FLAG-HrpB was unexpectedly detected in the supernatant fraction (Fig. 5B). To distinguish soluble supernatant proteins from those that may be associated with insoluble membrane components (e.g., outer membrane vesicles [OMVs]), we depleted insoluble components of the supernatant via ultracentrifugation and tested the clarified soluble fraction for Hrp proteins. Effectively all HrpB in the supernatant was removed with ultracentrifugation, suggesting it is localized in OMVs (Fig. 5C) (36). Also, in the absence of HrpB coexpression, the levels of HrpC detected in the supernatant were reduced upon removal of insoluble material, while supernatant levels of HrpC detected after coexpression with HrpB were not affected by removal of insoluble material (Fig. 5D). We conclude that HrpB is membrane bound and that it secretes soluble HrpC protein into the extracellular milieu.

### Host environment drives TXISS class.

**FIG 6:**
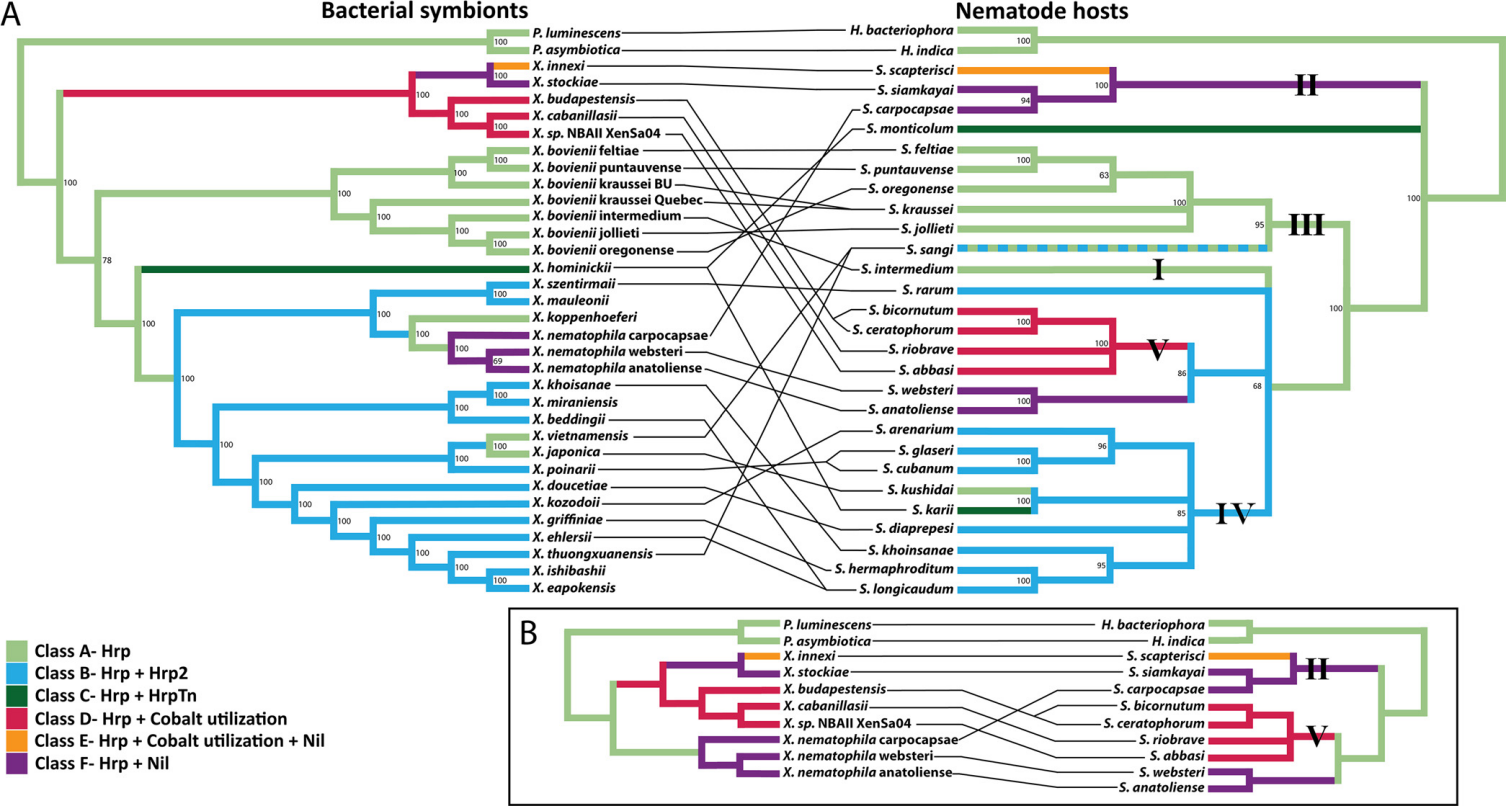
Cladograms of *Xenorhabdus* and *Steinernema* color coded according to *Xenorhabdus* DUF560 class. (A and B) Cophylogeny of nematode species and their colonizing bacteria across the *Steinernema* genus (A) or with a focus on specific clades (B). Numbers on branches indicate bootstrap support values. Bootstrap values below 60% were contracted. Lines connecting the phylogenies indicate mutualist pairs. Roman numerals highlight the five *Steinernema* clades described in reference 41. Colored overlays indicate the DUF560 class of a given bacterium or a given nematode’s symbiont as follows: class A (light green), class B (light blue), class C (dark green), class D (red), class E (orange), and class F (purple).

Having established that DUF560 homologs represent a bona fide secretion system, we next used the *Xenorhabdus* system to expand on our observation that the presence and type of TXISS correspond to environmental niche. *Xenorhabdus* bacteria are species-specific obligate mutualists of *Steinernema* nematodes, and NilB is a known host range determinant. Therefore, we considered host species as an environmental niche and bioinformatically examined whether the complement of DUF560 genes in a microbe corresponds with host phylogeny (37–41). All 46 *Xenorhabdus* genomes on the Magnifying Genome (MaGe) platform (42) encode between one and three TXISS, with five distinct homologs represented across the genus (one TXISS-1A and four TXISS-1B) (43). The one TXISS homolog that is conserved among all *Xenorhabdus* species is TXISS-1B HrpB. The genomic contexts of six representative homologs are shown in Fig. S4. Each unique combination of TXISS homologs was assigned one of six classes (A to F) (Fig. S4 and Table S3). To visualize correlations between TXISS class and host identity, we constructed maximum likelihood and Bayesian phylogenetic trees for *Xenorhabdus* and *Steinernema*. *Xenorhabdus* trees were generated with whole-genome data, while *Steinernema* trees used five available loci (Fig. S5 and Table S4). Aligning the maximum likelihood phylogenies reveals that the TXISS complement of a *Xenorhabdus* species is more predictive of nematode host than phylogenetic position (Fig. 6).

10.1128/mBio.01956-21.4 FIG S4 Representative genomes of *Xenorhabdus* DUF560 classes A to F. Schematic diagrams of *Xenorhabdus* TXISS loci representing each of the six classes defined in the text. One species from each of the classes was selected for presentation. Box arrows represent open reading frames (ORFs), which are color coded according to predicted annotated function as indicated by the legend. The DUF560 homolog is shown in red, and the predicted TXISS cargo is shown in orange. Large ORFs were not presented in their entirety, and the length of the gap is indicated above the break line shown within such ORFs. Download FIG S4, PDF file, 0.2 MB. Copyright © 2021 Grossman et al. 2021 Grossman et al. https://creativecommons.org/licenses/by/4.0/ This content is distributed under the terms of the Creative Commons Attribution 4.0 International license.

10.1128/mBio.01956-21.5 FIG S5 Bayesian posterior probability phylogenies. (A) Phylogram of select *Xenorhabdus* bacteria, based on concatenations of 665 conserved core genes. Numbers indicate posterior probability values. Distances indicate substitutions per base pair. (B) Bayesian phylogeny of select entomopathogenic nematodes, based on concatenations of the internal transcribed spacer (ITS), 18S rRNA, 28S rRNA, cytochrome oxidase C subunit I (COI), and 12S rRNA loci. Two members of the sister taxon *Photorhabdus* were chosen as an outgroup. Loci are recorded in Table S4. Download FIG S5, PDF file, 0.2 MB. Copyright © 2021 Grossman et al. 2021 Grossman et al. https://creativecommons.org/licenses/by/4.0/ This content is distributed under the terms of the Creative Commons Attribution 4.0 International license.

10.1128/mBio.01956-21.8 TABLE S3 *Xenorhabdus* DUF560 classes. Download Table S3, XLSX file, 0.4 MB. Copyright © 2021 Grossman et al. 2021 Grossman et al. https://creativecommons.org/licenses/by/4.0/ This content is distributed under the terms of the Creative Commons Attribution 4.0 International license.

10.1128/mBio.01956-21.9 TABLE S4 Locus tags used in phylogenetic analysis of *Xenorhabdus* and *Steinernema* species. Download Table S4, XLSX file, 0.2 MB. Copyright © 2021 Grossman et al. 2021 Grossman et al. https://creativecommons.org/licenses/by/4.0/ This content is distributed under the terms of the Creative Commons Attribution 4.0 International license.

This alignment of the phylogenetic placement of a given *Steinernema* host with the TXISS complement of the symbiont provides insights into the nematode internal environment experienced by the symbiont. For example, *Xenorhabdus* with two HrpB paralogs at the *hrp* locus are symbionts of nematodes within the phylogenetic clade IV, suggesting that these nematodes present a distinctive environment in which a second *hrp* locus is beneficial. *Xenorhabdus* with an HrpB paralog encoded adjacent to genes predicted to encode a cobalt ABC transporter are solely symbionts of clade V nematodes. Xenorhabdus innexi and Xenorhabdus stockiae have seemingly diverged from this lineage through acquisition of a *nilB* homolog and switching to hosts within clade II. Similarly, X. nematophila independently gained *nilB* and switched into a clade II host (Fig. 6B). These acquisitions, alongside the varied genomic contexts of *nilB*/*nilC* pairs, are consistent with previous suggestions that the *nil* locus was horizontally acquired among *Xenorhabdus* (18).

**FIG 7:**
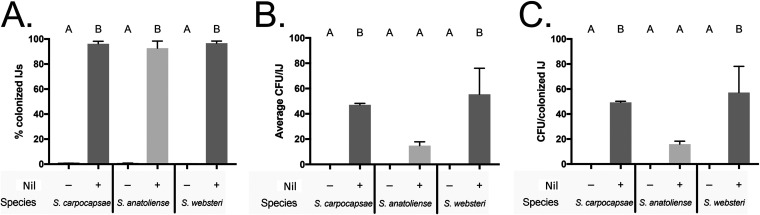
The *nil* locus is necessary for colonization of *S. anatoliense*, *S. carpocapsae*, and *S. websteri* infective juveniles. Bacterium-free *S. carpocapsae*, *S. anatoliense*, and *S. websteri* were exposed to GFP-expressing *X. nematophila* lacking or bearing the *nil* locus. (A and B) The resulting progeny infective juveniles (IJs) were monitored for colonization either by microscopy (A) or by plating lysates for average CFU/IJ. (B) (C) The average CFU per colonized IJ combines both of these values to show bacterial load per organism. Treatments were analyzed via one-way ANOVA and Tukey’s *post hoc* test.

The TXISS NilB and the lipoprotein NilC are necessary for *X. nematophila* to colonize the clade II nematode S. carpocapsae (14, 18). However, *X. nematophila* also colonizes two nematodes, Steinernema anatoliense and Steinernema websteri, which are phylogenetically separate from clade II (Fig. 6). Our hypothesis that TXISS are involved in host-environment adaptations leads to the prediction that *X. nematophila* will require the *nil* locus to colonize these nematodes. To test this hypothesis, bacterium-free S. anatoliense, S. websteri, and S. carpocapsae eggs (44) were exposed to an *X. nematophila* carpocapsae ATCC 19061 Δ*nil* mutant and a *nil*-complemented strain (15). Consistent with our prediction, the Δ*nil* mutant was deficient in infective juvenile colonization in all three nematode species (Fig. 7), demonstrating that *nil* genes are necessary for infective juvenile colonization of *S. anatoliense* and *S. websteri* and supporting our hypothesis that TXISS promote adaptation to host environments.

## DISCUSSION

Bacteria have evolved specialized secretion systems for delivery of effectors that facilitate the host-associated lifestyle. Knowledge of cargo protein identities and sorting processes facilitates predictions from genomic information of bacterial secretome composition, regulation, and localization. Despite the diverse secretion systems now recognized, more remain to be identified based on the fact that some proteins predicted to be secreted lack known secretion pathways (45). The type X secretory pathway was described in 2020 shedding light on long-standing mysteries surrounding the dependence on a muramidase for secretion of typhoid toxin across the cell wall (7). Enabled by availability of genomic data from myriad environments and ever-improving bioinformatic visualization tools, we have presented data that the TXISS is a broadly distributed molecular vehicle for moving proteins across the Gram-negative outer membrane. Our network analysis has revealed functionally relevant TXISS clusters, with cluster 1 members having a conserved role in host-microbe interactions.

The DUF560 (domain of unknown function) family presence in animal-associated bacteria was first recognized when it was noted that the host colonization factor NilB has homologs in several human pathogens (14). This observation was strengthened by subsequent demonstration that Slam1 and Slam2 from these bacteria facilitate surface presentation of host metal acquisition proteins (8, 9, 14). Using the *Steinernema-Xenorhabdus* symbiosis, here we provide evidence that the composition of TXISS in a bacterial symbiont genome correlates with host organism. During the evolutionary history of *Xenorhabdus*, the gain or loss of TXISS loci correlated with host switching events (Fig. 6). These data indicate that TXISS activity contributes to bacterial adaptation to new host environments, which is particularly relevant given the varied distribution of TXISS homologs among human pathogens. For instance, our work enables categorization of TXISS among animal-associated *Neisseria* species, including the human pathogens Neisseria meningitidis and Neisseria gonorrhoeae (46). *Neisseria* strains can encode up to 6 TXISS paralogs. N. meningitidis MC58 has two functionally characterized TXISS, Slam1 and Slam2. Our network analysis indicates N. meningitidis encodes a third Slam, NMB1466/NP_274965, that also falls within cluster 1A. N. gonorrhoeae TXISS are represented in more nodes than N. meningitidis, notably occupying 19.3% of nodes in subcluster 1B (Fig. 3). The cluster framework we present here indicates that *Neisseria* use TXISS to secrete both lipoproteins and soluble cargo and that variation in TXISS composition among *Neisseria* strains may be predictive of host or tissue association phenotypes.

While DUF560 proteins were originally thought to represent a mechanism for lipoprotein surface exposure (8), recent studies have expanded that functional range to include peripheral membrane proteins (11) and now soluble secreted proteins, establishing the function of DUF560 outer membrane proteins in secretion of varied substrates. We predict that further study will uncover even more chemically diverse cargo for the distinct classes of TXISS, revealed through our network analysis. Here we focused on a single cluster of a DUF560 sequence similarity network (SSN). The remaining nine clusters likely represent diverse subfamilies responsible for transporting as-yet-unknown cargo. The lipoprotein cargo proteins for which structural conformations are known, share a C-terminal eight-stranded β-barrel (9). Our discovery that HrpC is a cargo protein for TXISS-1B HrpB strengthens the concept that this barrel is an important characteristic of TXISS cargo; HrpC is a homolog of the H. haemolyticus hemophilin, the structure of which likewise adopts a C-terminal eight-stranded β-barrel (29) (see Fig. S2 in the supplemental material). These data support the concept that TXISS cargo have bifunctional structures in which the N terminus is the host effector domain, while the C terminus targets secretion. This framework will facilitate identification of as-yet-unknown TXISS cargo among the genes that co-occur with TXISS outer membrane proteins. It is our hope that the DUF560-TbpBBD co-occurrence table used for our network annotation will become a resource for other microbiologists studying secreted proteins (see Table S2 in the supplemental material). This database identifies 851 potential TXISS/cargo pairs spread across 463 bacterial isolates, only 4 of which have been experimentally investigated thus far. Furthermore, our analyses suggest that SSN clusters have predictive power for other characteristics of TXISS cargo, including whether they are surface attached or secreted. The network-enabled classification presented here will facilitate the investigation of both TXISS outer membrane proteins (OMPs) and their cargo in diverse bacteria.

Slam1 and Slam2 TXISS-dependent cargo with known molecular function include those that serve as coreceptors for TonB-dependent metal uptake systems (2). Similarly, HrpC, which we show here is a TXISS-dependent cargo protein, and its homologs also likely function in metal acquisition by acting as hemophores akin to hemophilin and HasA (29, 47, 48). This idea is supported by the fact that an N. gonorrhoeae *hrpC* homolog (NGO0554) is repressed by iron, upregulated in response to oxidative stress, and contributes to resistance to peroxide challenge (49–51). Further, the *hrp* locus, which is conserved across proteobacteria, is predicted to encode TonB and a TonB-dependent receptor (Fig. S4). Our working model is that the metal bound by secreted HrpC is passed to its respective TonB-dependent receptor and imported into the cell through TonB energization (2). Given the conservation of the TXISS *hrp* locus among all *Xenorhabdus* and throughout human microbiome constituents, it will be important in the future to examine the regulation of HrpC TXISS-dependent secretion and the roles of *hrp* machinery in binding and acquiring host metals in a mucosal environment.

In addition to their long-recognized roles in import, TonB and TonB-dependent receptors may also function in protein export. The Myxococcus xanthus TonB-dependent transporter Oar can export the protease PopC using the proton motive force to energize membrane translocation (52). Despite this expansion of the known functional range of TonB-dependent transporters, in the case of the TXISS clusters, TonB is likely responsible for energizing uptake systems, but not TXISS-mediated export. This suggestion is supported by the fact that both the TXISS-1A function of Slam-1 (8) and the TXISS-1B function of HrpB shown here could be reconstructed in E. coli without coexpression of native TonB. However, it will be interesting for future studies to investigate possible functional interactions between TonB-dependent transporter-mediated secretion and TXISS-mediated secretion.

Our initial network generated from the entire PF04575 (DUF560) data set included cluster 5, comprising 14 nodes of Klebsiella homologs and 2 nodes with Klebsiella and Escherichia homologs annotated as PgaA/HmsH. This cluster was removed based on its limited number of nodes, predicted topology differences relative to the rest of the network (16-stranded versus 14-stranded barrel), and the fact that the majority of known PgaA homologs were not represented within the cluster. PgaA is a component of one of several machineries for secretion of exopolysaccharides (EPSs) that comprise biofilm matrices of Gram-negative bacteria (53–55). Despite the topological and substrate (polysaccharide versus protein) differences, the PF04575 assignment of cluster 5 PgaA homologs hints that there could be evolutionary or structural parallels between exopolysaccharide synthase-dependent secretion systems and TXISS. For instance, both TXISS and EPS secretion machineries either have, or associate with proteins that have, tetratricopeptide repeat (TPR) domains (53). In EPS secretion systems, the TPR are necessary for secretion or for cargo modification (54, 56). TXISS TPR domains may similarly modulate activities of other proteins that influence secretion of TXISS substrates. Support for a role of TPR domains in TXISS activity comes from the fact that *X. nematophila* expressing versions of the NilB TPR domain with small deletions display colonization defects that are ameliorated by deletion of the entire N-terminal periplasmic domain (15). Our establishment of TXISS as a bona fide secretion system opens new avenues for exploration of its integration with other export and secretion machineries and its coordinated contributions to host-associated phenotypes, including metal homeostasis, aggregation, and biofilm formation.

## MATERIALS AND METHODS

All primers and strains are listed in Table S5 in the supplemental material.

10.1128/mBio.01956-21.10 TABLE S5 Primer and strain list. Download Table S5, XLSX file, 0.01 MB. Copyright © 2021 Grossman et al. 2021 Grossman et al. https://creativecommons.org/licenses/by/4.0/ This content is distributed under the terms of the Creative Commons Attribution 4.0 International license.

### DUF560 SSN analysis.

The EFI Enzyme Similarity Tool (EFI-EST) was used to collect all predicted DUF560 domain-containing sequences from Interpro 73 and UniProt 2019-02 (accessed 24 April 2019) and compare using BLAST (21, 22, 25). Representative networks collapsed nodes which shared ≥40% identity. On an EFI-EST network, edges are drawn according to a database-independent alignment score. Greater scores correspond to fewer edges. For separation of DUF560 clusters, an alignment score of 38 was chosen (Fig. 2). For subclusters 1A and 1B, 89 and 100 were chosen, respectively (see Fig. S1 in the supplemental material). Networks were visualized and interpreted using Cytoscape v3.7.1 (57) and Gephi v0.9.2 (58). Nodes were arranged with the Fruchterman-Reingold force-directed layout *algorithm* (59).

The contents of each cluster were compared to Pfam DUF560 (PF04575) to ensure that clusters contained legitimate DUF560 proteins (26). Any clusters for which fewer than 18% of sequences were present in Pfam, or with fewer than 20 sequences, were excluded. This filtering removed cluster 5, composed mostly of Klebsiella pneumoniae PgaA. The resulting SSN contains 10 clusters, 1,222 nodes, and 52,190 edges with 1,589 TaxIDs represented (Fig. 2). Using NCBI Taxonomy Browser, each node was manually curated for the isolate’s environmental origin(s) among the following categories: water, soil, plant, mammal, animal, invertebrate, nematode, built (environments such as sewage, bioreactors, etc.), multiple environments, and unclassified (Table S1). If no citation was available, the source was curated from BioSample or BioProject records or if neither was available, from other resources (NCBI linkout, Google search). Animal-associated microbes were designated as mammal, insect, nematode, or generic animal associated. Our fine-scale analysis focused on cluster 1 and its subclusters (Fig. 3 and Fig. S1).

Three different techniques were used to determine whether DUF560 proteins within our network were genomically associated with TbpBBD domain-containing proteins. First, using EFI-GNN, genome-neighborhood-networks were generated for subcluster 1A (alignment score, 89; 40 total flanking ORFs, 20 upstream and 20 downstream of the DUF560), subcluster 1B (alignment score, 38; 20 total flanking ORFs, 10 upstream and 10 downstream of the DUF560), and subcluster 1C (alignment score, 38; 20 total flanking ORFs, 10 upstream and 10 downstream of the DUF560), resulting in 352 DUF560-TbpBBD pairs (22). Next, the RODEO web tool was used to analyze cooccurrence using profile hidden Markov models to assign domains to local ORFs resulting in 712 DUF560-TbpBBD pairs (27). Seven additional DUF560-TbpBBD pairs in *Xenorhabdus* were manually annotated. All three data sets were combined for a total of 851 nonredundant protein pairs (Table S2). SignalP-5 (60, 61) was used to predict the signal peptides of all TbpBBD domain-containing proteins and annotate each node (Fig. 3C). Any node associated with both signal peptide-bearing and signal peptide-free proteins was annotated according to those with a signal.

### DUF560 genome neighborhood analysis.

Subclusters 1A to C were separated and analyzed in EFI-EST with an alignment score of 38 as described above. Each network was then analyzed through EFI-GNN and visualized in Cytoscape v3.7.1 (57) (alignment score, 38; 10 ORFs up- and downstream).

### Phyre^2^ analysis.

After removal of signal peptide sequences, TbpBBD_sol_ proteins *X. nematophila* HrpC, P. rettgeri PROVETT_08181 and PROVETT_05852, and Proteus mirabilis WP_134940027.1 were submitted to Phyre^2^ Protein Homology/analogy Recognition Engine v.2.0 to predict potential three-dimensional (3-D) structures (32). The top predicted match for each, hemophilin (PDB accession no. 6OM5), was used to generate structural models (Fig. S2).

### Expression of HrpB and HrpC.

The gene encoding HrpB25_26insDYKDDDDK (FLAG-HrpB) was synthesized and cloned into the second multiple cloning site (MCS) of pETDuet-1 by Genscript. The genomic region containing HrpC was amplified from the *X. nematophila* ATCC 19061 (HGB800) genome using primers 1 and 2, digested with restriction enzymes SacI and SalI, and ligated into the pUC19 MCS. Site-directed mutagenesis was used to add a C-terminal FLAG tag onto HrpC using primers 3 and 4. The gene encoding HrpC1_2insV246_247insDYKDDDDK (henceforth HrpC-FLAG) was amplified from pUC19 using primers 5 and 6. The product was ligated into MCS1 of both pETDuet-1 and pETDuet-1/FLAG-HrpB, resulting in expression plasmids pETDuet-1/HrpC-FLAG and pETDuet-1/HrpC-FLAG/FLAG-HrpB. HrpC-FLAG was used to create HrpC1_2insV246_247ins3x(GGGGS)-GST domain (HrpC-GST) using Hi-Fi assembly and primer pair 7 and 8 and primer pair 9 and 10 to integrate a 15-amino-acid linker and GST into the previous FLAG locus. This process yielded pETDuet-1/HrpC-GST and pETDuet-1/HrpC-GST/FLAG-HrpB. All clones were confirmed by Sanger sequencing using primers 11 and 14 at the Univeriity of Tennessee (UT) Genomics Core.

Expression plasmids were transformed into E. coli B21(DE3) and E. coli C43(DE3) via electroporation. Results were similar for the two strains. Strains were grown in defined medium with 150 μg/ml ampicillin (62). Bacteria were subcultured into 100 ml of broth at an initial optical density at 600 nm (OD_600_) of 0.028, grown for 18 h at 37°C to reach late logarithmic growth, and induced with 500 μM isopropyl-β-d-thiogalactopyranoside (IPTG). After 1 h, 700 μl of each culture was filtered for subsequent use. At 2.5 h, whole cells were collected by centrifugation and lysed using a bead beater. Remaining supernatants were filtered. Protein concentration of whole-cell lysates was measured via Bradford assay. For supernatant samples, 700 μl of supernatant was precipitated via 10% trichloroacetic acid (TCA) (63).

Samples were analyzed by 10% sodium dodecyl sulfate-polyacrylamide gel electrophoresis (SDS-PAGE) and immunoblotting. For lysates, wells were loaded with 9.5 μg of protein. For supernatants, wells were loaded with half the TCA precipitate collected media. Western blots were probed with either rat anti-FLAG or rabbit anti-GST primary antibody anti-IgG secondary antibody that fluoresces at 680 nm. Intensities were recorded for FLAG or GST reactive bands which were the correct size for mature HrpB and HrpC. For every supernatant sample, a distinct band from the Coomassie blue-stained gel was used as a loading control to normalize intensities of supernatant samples prior to analysis (Fig. S3). Unpaired *t* tests were used to compare HrpC-FLAG to HrpC-GST secretion and supernatant fraction HrpB. One replicate of the HrpC-GST 1-h supernatant was excluded from analysis due to cellular lysis. A Tukey’s honestly significant difference (HSD) test was used for comparing supernatant fraction HrpC.

For additional analysis of OMVs, bacteria producing each expression plasmid were subcultured in triplicate into 40 ml of broth at an initial OD_600_ of 0.04. After 5 h at 37°C to reach mid-logarithmic growth, cultures were induced with 500 μM IPTG. After an additional 2.5 h, each culture was clarified via centrifugation, then the supernatant was filtered and centrifuged at 150,000 relative centrifugal force (RCF) for 3 h. The entire OMV pellet was solubilized in SDS sample buffer. Six hundred microliters of the total supernatant or the clarified (postultracentrifugation) supernatant were precipitated via 10% TCA and resuspended in SDS sample buffer (63). Samples were analyzed by SDS-PAGE and Western blotting as described above.

### Phylogenetic tree generation.

Phylogenetic analysis was performed as described previously (64). Briefly, select *Xenorhabdus* and *Photorhabdus* species were analyzed using MicroScope MaGe’s Gene Phyloprofile tool (37, 42) to identify homologous protein sets which were conserved across all assayed genomes. Putative paralogs were excluded from downstream analysis to ensure homolog relatedness, resulting in 665 homologous sets (Table S5). Homolog sets were retrieved via locus tag indexing using BioPython (65), individually aligned using Muscle v3.8.31 (66), concatenated using Sequence Matrix v1.8 (67), and trimmed of nucleotide gaps using TrimAL v1.3 (68). JmodelTest v2.1.10 (69) was used to choose the GTR+γ substitution model for maximum likelihood and Bayesian analysis.

For nematode phylogenetic analysis, select *Steinernema* and *Heterorhabditis* species were analyzed. The internal transcribed spacer, 18S rRNA, 28S rRNA, cytochrome oxidase I, and 12S rRNA loci were collected from GenBank and used as homologous sets (Table S4). Nematode species with fewer than three of five loci sequenced were excluded. Homologous sets were processed using the same methods as the *Xenorhabdus* sequences, although for these sequences Modettes v2.1.10 (70) chose the GTR+γ+I substitution model.

Maximum likelihood analyses were performed via RAxML v8.2.10 (70) using rapid bootstrapping and 1,000 replicates and were visualized via Dendroscope v3.6.2 (71). Nodes with less than 60% bootstrap support were collapsed. Bayesian analyses were performed via MrBayes v3.2.6 with BEAGLE (72–74) on the CIPRES Science Gateway platform (75). A total of 500,000 or 4,000,000 Markov chain Monte Carlo (MCMC) replicates were performed for the bacterial or nematode tree, respectively. Twenty-five percent were discarded as burn-in, and posterior probabilities were sampled every 500 replicates. Two runs were performed with three heated chains and one cold chain. The final standard deviation of split frequencies was less than 10^−6^ for the bacterial tree, and it was 0.002557 for the nematode tree. Bayesian trees were visualized with FigTree v1.4.4 (76). Bayesian and maximum likelihood methods generated phylogenies with consistent topologies (Fig. 6 and Fig. S5).

### Wild type versus Δ*nil* colonization of nematodes.

Bacterium-free eggs of S. anatoliense, S. carpocapsae, and S. websteri (44) exposed to a green fluorescent protein (GFP)-expressing Nil mutant (HGB1495) or its complemented strain (HGB1496) were grown on lipid agar plates for 2 days at 25°C (15). Plates were placed into White traps 1 week after plating to collect infective juvenile (IJ) nematodes. Nematode colonization was visualized using fluorescence microscopy on a Keyence BZX-700 to observe bacteria in the receptacle (in biological triplicate and technical duplicate). To determine the number of CFU per IJ, nematodes were surface sterilized and ground for 2 min using a Fisher brand motorized tissue grinder (catalog no. 12-1413-61) to homogenize the nematodes and release colonizing bacteria. Serial dilutions in phosphate-buffered saline (PBS) were plated on LB agar and then incubated at 30°C for 1 day before enumerating CFU (Fig. 7). To calculate the CFU per colonized IJ, the percent colonized nematodes was divided by the CFU/IJ for each biological replicate. The data were analyzed using a one-way analysis of variance (ANOVA) with Tukey’s multiple-comparison test to compare the mean of each treatment.
