# Supplementary material for: A Widespread Bacterial Secretion System with Diverse Substrates
Source: mBio. 2021 Aug 17;12(4):e01956-21. doi: 10.1128/mBio.01956-21 (PMC8406197; doi:10.1128/mBio.01956-21)
Supplement: FIG S1 [file mbio.01956-21-sf001.pdf]

A Widespread Bacterial Secretion System with Chemically Diverse Protein Substrates

Alex S. Grossman[a¶], Terra J. Mauer[b]*, Katrina T. Forest[b], and Heidi Goodrich-Blair[a,b]#
[a]University of Tennessee-Knoxville, Department of Microbiology, Knoxville, TN
[b]University of Wisconsin-Madison, Department of Bacteriology, Madison, WI
#Address correspondence to Heidi Goodrich-Blair, hgblair@utk.edu

Supplementary Figure 1

FIG S1 Increased stringency separates subcluster 1A and 1B into functional groups. A series of stringent EFI-EST sequence similarity networks highlights detail in cluster 1 of the DUF560 homologs. Edge darkness demonstrates similarity. Node positioning was optimized using the Fruchterman-Reingold algorithm (4). Dotted lines indicate hypothetical functional clusters based on previous molecular data. Circled nodes indicate proteins which have been molecularly characterized. Subclusters 1A and 1B were analyzed separately to allow fine tuning of alignment score (89 and 100 respectively). Networks were color coded to display either taxonomic categories or co-inheritance with TbpBBD-domain containing proteins.

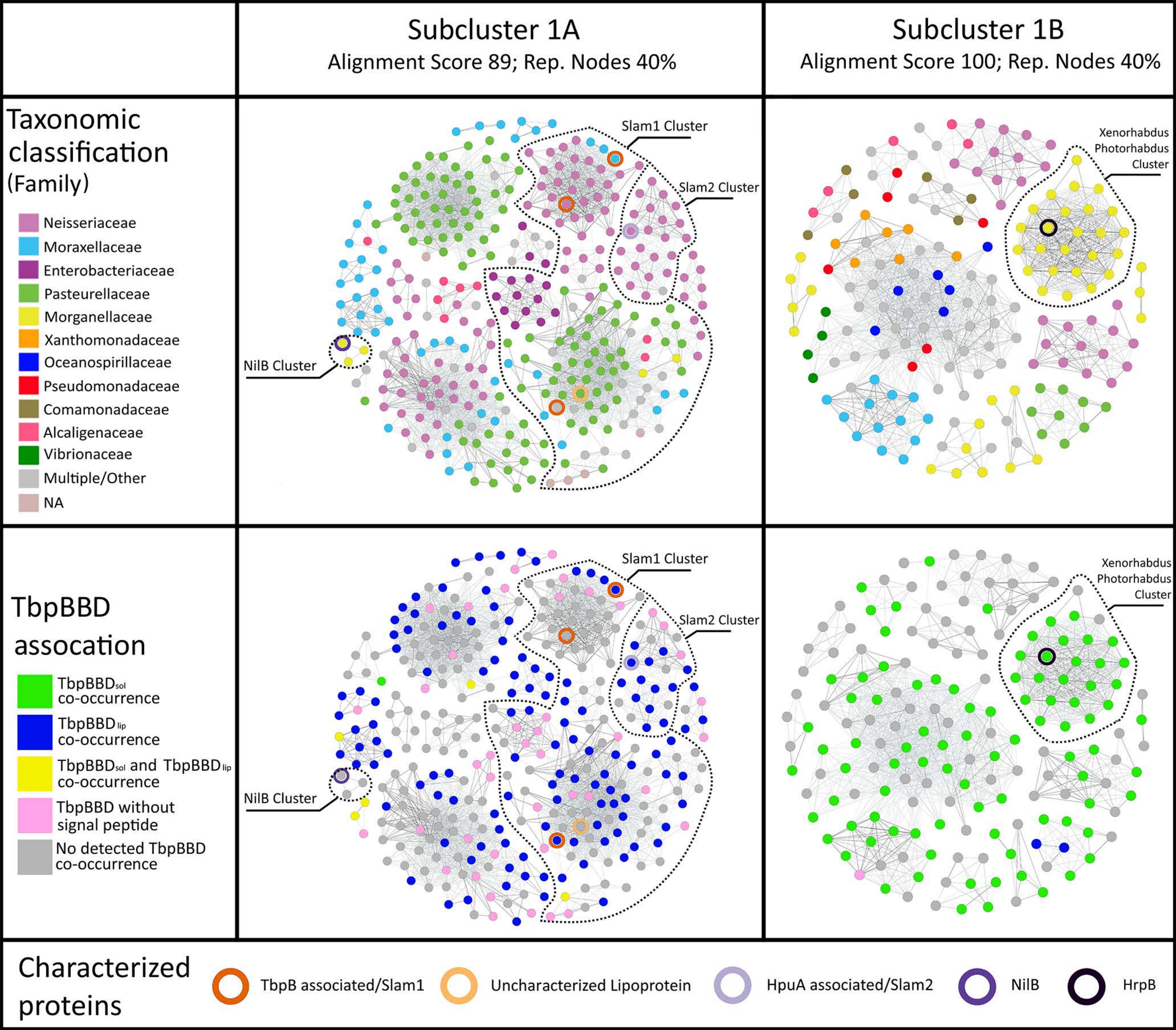

Subcluster 1A
Alignment Score 89; Rep. Nodes 40%
Subcluster 1B
Alignment Score 100; Rep. Nodes 40%
Taxonomic classification (Family)
Neisseriaceae
Moraxellaceae
Enterobacteriaceae
Pasteurellaceae
Morganellaceae
Xanthomonadaceae
Oceanospirillaceae
Pseudomonadaceae
Comamonadaceae
Alcaligenaceae
Vibrionaceae
Multiple/Other
NA
Slam1 Cluster
Slam2 Cluster
NilB Cluster
Xenorhabdus Photorhabdus Cluster
TbpBBD assocation
TbpBBDsol co-occurrence
TbpBBDlip co-occurrence
TbpBBDsol and TbpBBDlip co-occurrence
TbpBBD without signal peptide
No detected TbpBBD co-occurrence
Slam1 Cluster
Slam2 Cluster
NilB Cluster
Xenorhabdus Photorhabdus Cluster
Characterized proteins
TbpB associated/Slam1
Uncharacterized Lipoprotein
HpuA associated/Slam2
NilB
HrpB
