# Supplementary material for: A Widespread Bacterial Secretion System with Diverse Substrates
Source: mBio. 2021 Aug 17;12(4):e01956-21. doi: 10.1128/mBio.01956-21 (PMC8406197; doi:10.1128/mBio.01956-21)
Supplement: FIG S2 [file mbio.01956-21-sf002.pdf]

A Widespread Bacterial Secretion System with Chemically Diverse Protein Substrates

Alex S. Grossman[a¶], Terra J. Mauer[b*], Katrina T. Forest[b], and Heidi Goodrich-Blair[a,b#]
[a]University of Tennessee-Knoxville, Department of Microbiology, Knoxville, TN
[b]University of Wisconsin-Madison, Department of Bacteriology, Madison, WI
#Address correspondence to Heidi Goodrich-Blair, hgblair@utk.edu

Supplementary Figure 2

FIG S2 Phyre[2] models of select $\text{TbpBBD}_{\text{sol}}$ proteins. $\text{TbpBBD}_{\text{sol}}$ proteins, lacking the signal sequence (-SS), from *X. nematophila* (HrpC), *P. rettgeri* (PROVRETT_08181 and 05852), and *P. mirabilis* (WP_134940027.1) were queried through the Phyre[2] Protein Homology/analogy Recognition Engine v. 2.0 (http://www.sbg.bio.ic.ac.uk/phyre2/html/page.cgi?id=index) (33). The top predicted structural model output for each is shown alongside the solved crystal structure of hemophilin from *H. haemolyticus* (protein data bank file 6OM5) which the algorithm selected as the template for all queries (29). PDB files were visualized with Protean 3D v15. (Protean 3D®. Version 15.0. DNASTAR. Madison, WI).

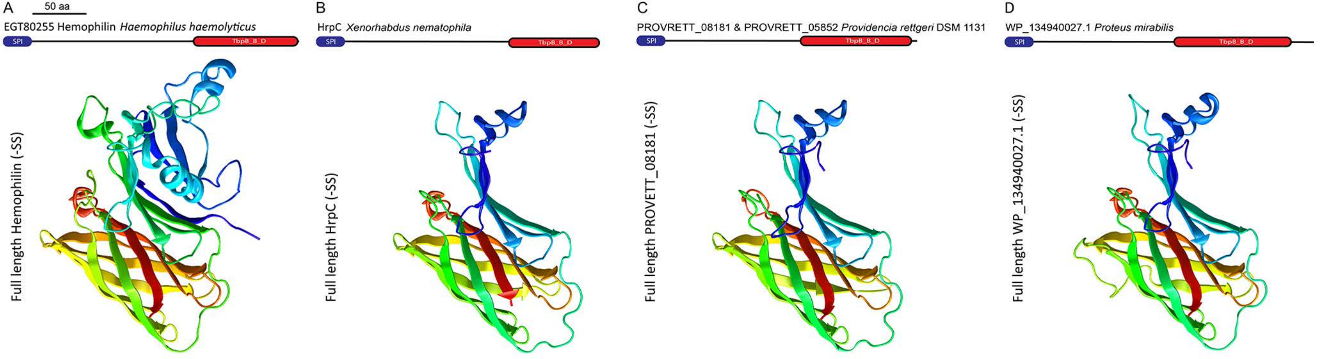

A
50 aa
EGT80255 Hemophilin *Haemophilus haemolyticus*
SPI
TbpB_B_D
Full length Hemophilin (-SS)
B
HrpC *Xenorhabdus nematophila*
SPI
TbpB_B_D
Full length HrpC (-SS)
C
PROVRETT_08181 & PROVRETT_05852 *Providencia rettgeri* DSM 1131
SPI
TbpB_B_D
Full length PROVETT_08181 (-SS)
D
WP_134940027.1 *Proteus mirabilis*
SPI
TbpB_B_D
Full length WP_134940027.1 (-SS)
