# Supplementary material for: A Widespread Bacterial Secretion System with Diverse Substrates
Source: mBio. 2021 Aug 17;12(4):e01956-21. doi: 10.1128/mBio.01956-21 (PMC8406197; doi:10.1128/mBio.01956-21)
Supplement: FIG S4 [file mbio.01956-21-sf004.pdf]

A Widespread Bacterial Secretion System with Chemically Diverse Protein Substrates

Alex S. Grossman[a¶], Terra J. Mauer[b]*, Katrina T. Forest[b], and Heidi Goodrich-Blair[a,b]#
[a]University of Tennessee-Knoxville, Department of Microbiology, Knoxville, TN
[b]University of Wisconsin-Madison, Department of Bacteriology, Madison, WI
#Address correspondence to Heidi Goodrich-Blair, hgblair@utk.edu

Supplementary Figure 4

FIG S4 Representative genomes of *Xenorhabdus* DUF560 Classes A-F. Schematic diagrams of *Xenorhabdus* TXISS loci representing each of the six classes defined in the text. One species from each of the classes was selected for presentation. Box arrows represent open reading frames (ORFs), which are color coded according to predicted annotated function as indicated by the legend. The DUF560 homolog is shown in red and the predicted TXISS cargo is shown in orange. Large ORFs were not presented in their entirety and the length of the gap is indicated above the break line shown within such ORFs.

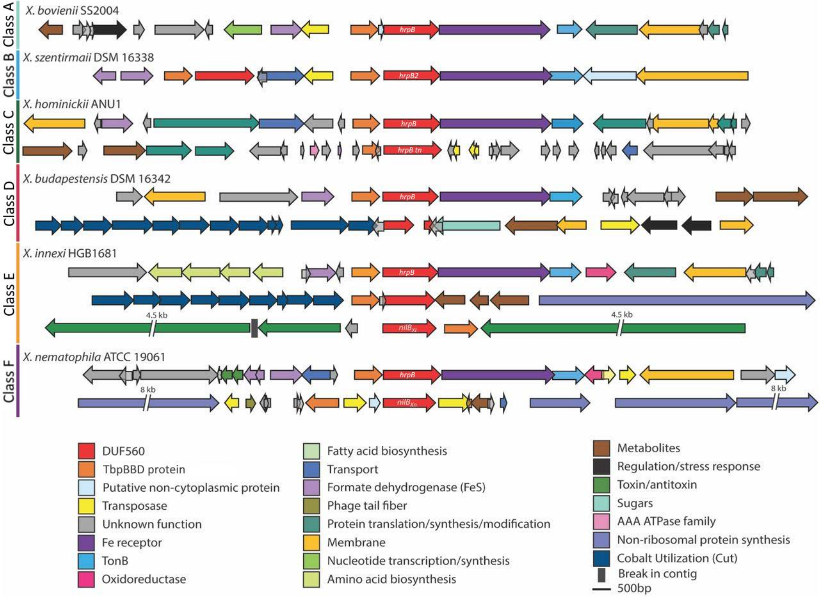

Class A
X. bovienii SS2004
hrpB
Class B
X. szentirmaii DSM 16338
hrpB2
Class C
X. hominickii ANU1
hrpB
hrpB tn
Class D
X. budapestensis DSM 16342
hrpB
Class E
X. innexi HGB1681
hrpB
4.5 kb
$nilB_{Xi}$
4.5 kb
Class F
X. nematophila ATCC 19061
hrpB
8 kb
$nilB_{Xn}$
8 kb
DUF560
TbpBBD protein
Putative non-cytoplasmic protein
Transposase
Unknown function
Fe receptor
TonB
Oxidoreductase
Fatty acid biosynthesis
Transport
Formate dehydrogenase (FeS)
Phage tail fiber
Protein translation/synthesis/modification
Membrane
Nucleotide transcription/synthesis
Amino acid biosynthesis
Metabolites
Regulation/stress response
Toxin/antitoxin
Sugars
AAA ATPase family
Non-ribosomal protein synthesis
Cobalt Utilization (Cut)
Break in contig
500bp
