# Supplementary material for: A Widespread Bacterial Secretion System with Diverse Substrates
Source: mBio. 2021 Aug 17;12(4):e01956-21. doi: 10.1128/mBio.01956-21 (PMC8406197; doi:10.1128/mBio.01956-21)
Supplement: FIG S5 [file mbio.01956-21-sf005.pdf]

A Widespread Bacterial Secretion System with Chemically Diverse Protein Substrates

Alex S. Grossman[a¶], Terra J. Mauer[b*], Katrina T. Forest[b], and Heidi Goodrich-Blair[a,b#]
[a]University of Tennessee-Knoxville, Department of Microbiology, Knoxville, TN
[b]University of Wisconsin-Madison, Department of Bacteriology, Madison, WI
#Address correspondence to Heidi Goodrich-Blair, hgblair@utk.edu

Supplementary Figure 5

FIG S5 Bayesian posterior probability phylogenies. (A) Phylogram of select *Xenorhabdus* bacteria, based on concatenations of 665 conserved core genes. Numbers indicate posterior probability values. Distances indicate substitutions per base pair. (B) Bayesian phylogeny of select entomopathogenic nematodes, based on concatenations of the ITS, 18S rRNA, 28S rRNA, COI, and 12S rRNA loci. Two members of the sister taxon *Photorhabdus* were chosen as an outgroup. Loci are recorded in Table S4.

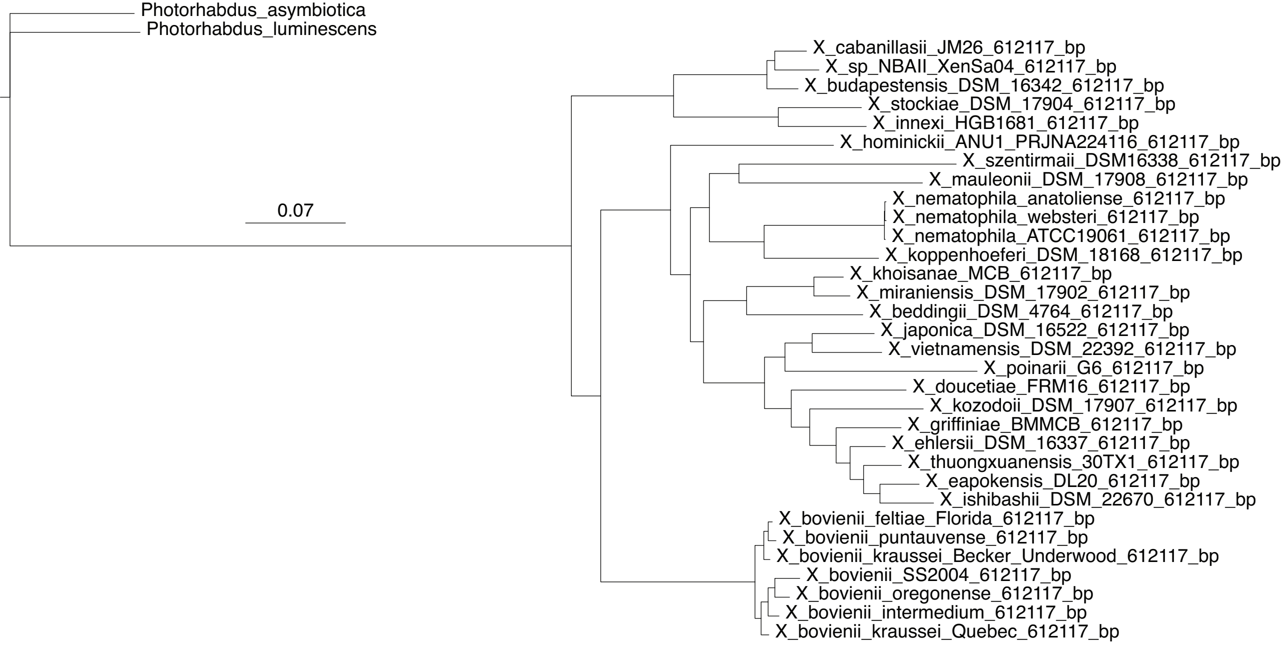
Photorhabdus_asymbiotica
Photorhabdus_luminescens
0.07
X_cabanillasii_JM26_612117_bp
X_sp_NBAII_XenSa04_612117_bp
X_budapestensis_DSM_16342_612117_bp
X_stockiae_DSM_17904_612117_bp
X_innexi_HGB1681_612117_bp
X_hominickii_ANU1_PRJNA224116_612117_bp
X_szentirmaii_DSM16338_612117_bp
X_mauleonii_DSM_17908_612117_bp
X_nematophila_anatoliense_612117_bp
X_nematophila_websteri_612117_bp
X_nematophila_ATCC19061_612117_bp
X_koppenhoeferi_DSM_18168_612117_bp
X_khoisanae_MCB_612117_bp
X_miraniensis_DSM_17902_612117_bp
X_beddingii_DSM_4764_612117_bp
X_japonica_DSM_16522_612117_bp
X_vietnamensis_DSM_22392_612117_bp
X_poinarii_G6_612117_bp
X_doucetiae_FRM16_612117_bp
X_kozodoii_DSM_17907_612117_bp
X_griffiniae_BMMCB_612117_bp
X_ehlersii_DSM_16337_612117_bp
X_thuongxuanensis_30TX1_612117_bp
X_eapokensis_DL20_612117_bp
X_ishibashii_DSM_22670_612117_bp
X_bovienii_feltiae_Florida_612117_bp
X_bovienii_puntauvense_612117_bp
X_bovienii_kraussei_Becker_Underwood_612117_bp
X_bovienii_SS2004_612117_bp
X_bovienii_oregonense_612117_bp
X_bovienii_intermedium_612117_bp
X_bovienii_kraussei_Quebec_612117_bp

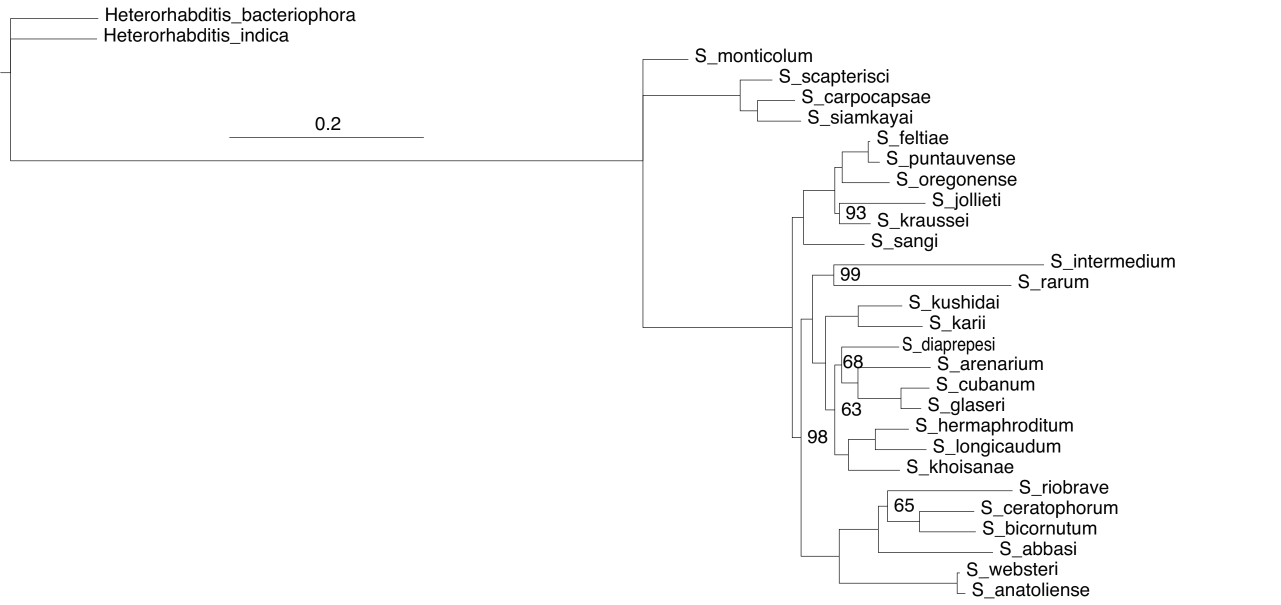
Heterorhabditis_bacteriophora
Heterorhabditis_indica
0.2
S_monticolum
S_scapterisci
S_carpocapsae
S_siamkayai
S_feltiae
S_puntauvense
S_oregonense
S_jollieti
93
S_kraussei
S_sangi
S_intermedium
99
S_rarum
S_kushidai
S_karii
S_diaprepesi
68
S_arenarium
S_cubanum
S_glaseri
63
S_hermaphroditum
98
S_longicaudum
S_khoisanae
S_riobrave
65
S_ceratophorum
S_bicornutum
S_abbasi
S_websteri
S_anatoliense
