# Supplementary material for: A Widespread Bacterial Secretion System with Diverse Substrates
Source: mBio. 2021 Aug 17;12(4):e01956-21. doi: 10.1128/mBio.01956-21 (PMC8406197; doi:10.1128/mBio.01956-21)
Supplement: FIG S3 [file mbio.01956-21-sf003.pdf]

A Widespread Bacterial Secretion System with Chemically Diverse Protein Substrates

Alex S. Grossman[a¶], Terra J. Mauer[b]*, Katrina T. Forest[b], and Heidi Goodrich-Blair[a,b]#
[a]University of Tennessee-Knoxville, Department of Microbiology, Knoxville, TN
[b]University of Wisconsin-Madison, Department of Bacteriology, Madison, WI
#Address correspondence to Heidi Goodrich-Blair, hgblair@utk.edu

Supplementary Figure 3

FIG S3 Western blots and Coomassie stained SDS-PAGE gels demonstrating secretion. Units to the left of each blot/gel are in kilodaltons (kDa). All experiments were repeated in triplicate as labeled above the lanes. Orange boxes in an immunoblot indicate either FLAG-HrpB, HrpC-FLAG, or HrpC-GST. Orange boxes on a Coomassie indicate the bands used for normalization of fluorescent intensity values. The top row shows Western blots of FLAG-HrpB/HrpC-FLAG co-expression probed with α-FLAG antibody and accompanying Coomassie stained SDS-PAGE gels. The ~50 kDa bands show FLAG-HrpB while the ~30 kDa bands show HrpC-FLAG. Expression of HrpB did not cause cell lysis or nonspecific protein export, if it had the banding pattern of the supernatant would look more like that of the lysates. The second row shows Western blots of FLAG-HrpB/HrpC-GST co-expression probed with α-GST antibody and Coomassie stained SDS-PAGE gels. The ~55 kDa bands show mature HrpC-GST while the lower bands are likely degradation products. The third row shows Western blots of FLAG-HrpB/HrpC-FLAG supernatant fractions probed with α-FLAG antibody and the accompanying Coomassie stained SDS-PAGE gels. The ~50 kDa FLAG-HrpB band is removed by ultracentrifuging the supernatants, demonstrating that the HrpB is likely in OMVs. The fourth row depicts a Western blot of the FLAG-HrpB/HrpC-FLAG OMV fraction probed with α-FLAG antibody and the accompanying Coomassie stained SDS-PAGE gel. OMVs can contain both FLAG-HrpB and HrpC-FLAG.

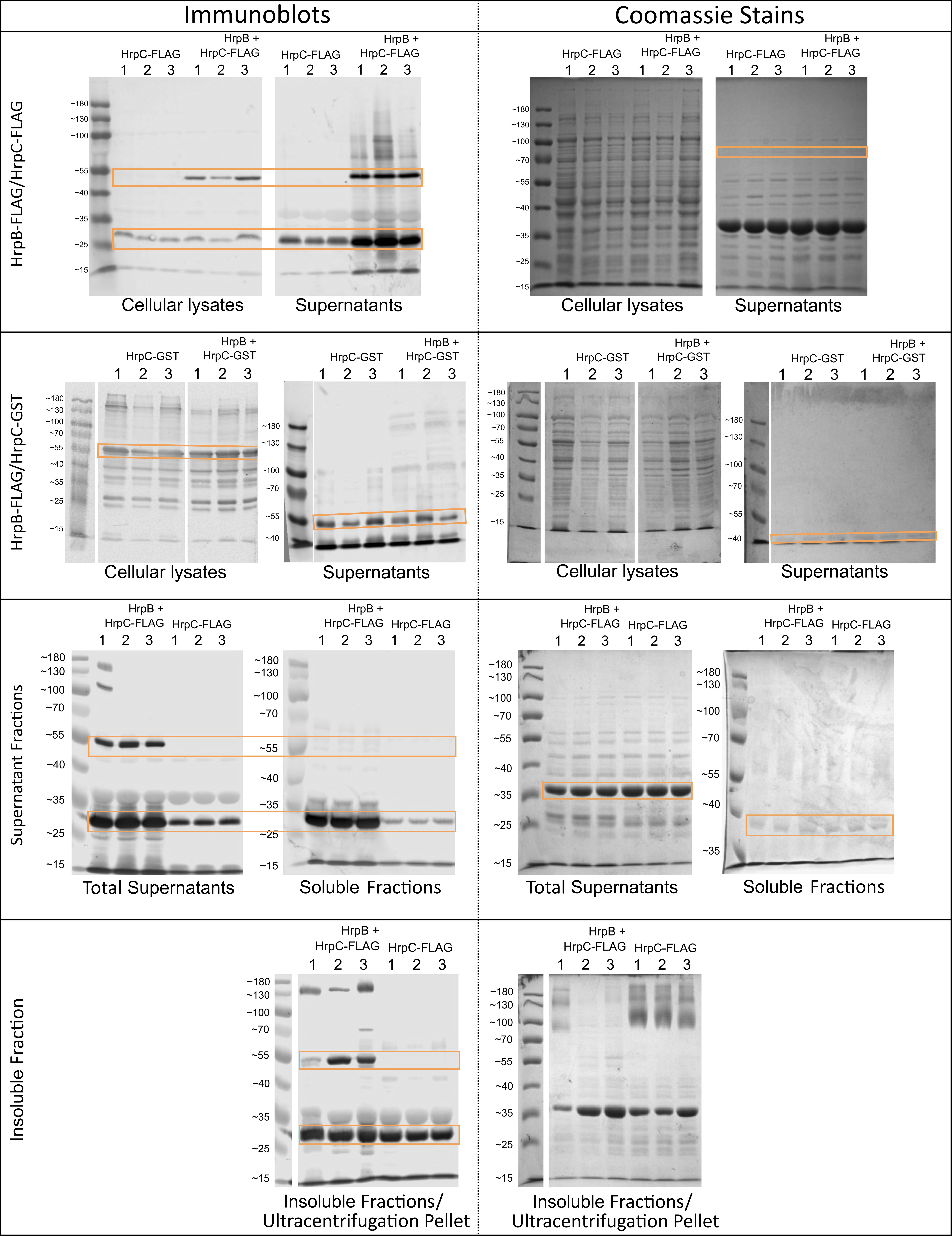

Immunoblots
Coomassie Stains
HrpB-FLAG/HrpC-FLAG
HrpC-FLAG
HrpB + HrpC-FLAG
1 2 3 1 2 3
~180
~130
~100
~70
~55
~40
~35
~25
~15
Cellular lysates
Supernatants
HrpB-FLAG/HrpC-GST
HrpC-GST
HrpB + HrpC-GST
Supernatant Fractions
Total Supernatants
Soluble Fractions
Insoluble Fraction
Insoluble Fractions/ Ultracentrifugation Pellet
